# Coupled Ferroelectric‐Photonic Memory in a Retinomorphic Hardware for In‐Sensor Computing

**DOI:** 10.1002/advs.202303447

**Published:** 2024-01-17

**Authors:** Ngoc Thanh Duong, Yufei Shi, Sifan Li, Yu‐Chieh Chien, Heng Xiang, Haofei Zheng, Peiyang Li, Lingqi Li, Yangwu Wu, Kah‐Wee Ang

**Affiliations:** ^1^ Department of Electrical and Computer Engineering National University of Singapore 4 Engineering Drive 3 Singapore 117583 Singapore

**Keywords:** convolution image processing, ferroelectric semiconductors, in‐sensor computing, optoelectronic memory, retinomorphic sensors

## Abstract

The development of all‐in‐one devices for artificial visual systems offers an attractive solution in terms of energy efficiency and real‐time processing speed. In recent years, the proliferation of smart sensors in the growth of Internet‐of‐Things (IoT) has led to the increasing importance of in‐sensor computing technology, which places computational power at the edge of the data‐flow architecture. In this study, a prototype visual sensor inspired by the human retina is proposed, which integrates ferroelectricity and photosensitivity in two‐dimensional (2D) α‐In_2_Se_3_ material. This device mimics the functions of photoreceptors and amacrine cells in the retina, performing optical reception and memory computation functions through the use of electrical switching polarization in the channel. The gate‐tunable linearity of excitatory and inhibitory functions in photon‐induced short‐term plasticity enables to encode and classify 12 000 images in the Mixed National Institute of Standards and Technology (MNIST) dataset with remarkable accuracy, achieving ≈94%. Additionally, in‐sensor convolution image processing through a network of phototransistors, with five convolutional kernels electrically pre‐programmed into the transistors is demonstrated. The convoluted photocurrent matrices undergo straightforward arithmetic calculations to produce edge and feature‐enhanced scenarios. The findings demonstrate the potential of ferroelectric α‐In_2_Se_3_ for highly compact and efficient retinomorphic hardware implementation, regardless of ambipolar transport in the channel.

## Graphical abstract

This work demonstrates a retinomorphic prototype that integrates ferroelectricity and photosensitivity within a two‐dimensional (2D) α‐In_2_Se_3_ material, emulating simultaneous functions in the human retina, that is, perceptive light‐sensing, memory, and computation. The optoelectronic memory efficiently classifies 12 000 images with a satisfactory precision of 94% and replicates five convolutional kernels using a network of 3 × 3 phototransistors for the image processing task.

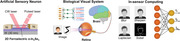

## 1 Introduction

Advances in light‐sensing technology pave the way for developing more sophisticated and intelligent devices, such as those employing in‐sensor computing techniques.^[^ ^1^, ^2^ ^]^ Conventional chips in complementary metal‐oxide‐semiconductor (CMOS) technology, on the other hand, are designed to capture the signal and convert them to back‐end digital data for processing by central processing units (CPU).^[^ ^3^ ^]^ Generally, this computing architecture requires separating multiple physical hardware, deteriorating energy efficiency, and slowing overall processing speed.^[^ ^1^, ^4^ ^]^ To address these issues, the in‐sensor computing paradigm has emerged, in which a sensory array performs some amount of computation on the data it captures before transferring it to a higher‐level processing unit.^[^ ^1^, ^5^, ^6^ ^]^ By integrating computing power into the sensor where the data is generated, in‐sensor computing allows the device to significantly reduce the amount of data and filter out the redundant or noisy information to extract essential features before sending it to post‐processing units. For instance, the common task in computer vision, e.g., autonomous driving, drones, and robotics, is object tracking, which only requires edge pixels of objects.^[^ ^5^, ^7^, ^8^ ^]^ Therefore, by incorporating edge enhancement directly within the sensor relative to the background, a more efficient solution can be achieved in terms of energy consumption and network latency.^[^ ^3^, ^9^, ^10^, ^11^ ^]^

Toward the competitive growth of artificial visual systems, retina‐inspired (retinomorphic) sensors that mimic the human retina's structure and function become a core technology for enhancing the efficiency of photoreception, memory, and computation for diverse Internet‐of‐Things (IoT) hardware.^[^ ^12^ ^]^ Promising studies have been conducted on retinomorphic devices for visual sensors, utilizing materials such as two‐dimensional (2D) WSe_2_,^[^ ^2^, ^10^, ^13^ ^]^ PtSe_2_,^[^ ^14^ ^]^ MoTe_2_/PdSe_2_ heterostructure,^[^ ^11^ ^]^ and electrostatically doped silicon,^[^ ^3^ ^]^ which exploit the natural ambipolar transport properties of these materials.^[^ ^15^ ^]^ In efforts to emulate the function of biological rod and cone cells in photoreceptors, oxide‐induced trap MoS_2_ photodiode^[^ ^16^ ^]^ and perovskite‐based homogeneous integration^[^ ^17^ ^]^ are employed to enable effective adaptation when processing information under dim or bright light. However, due to the lack of nonvolatile electrical switching resistance in channel materials, these device architectures required in situ electrostatic gating during the data collection process, which consumes tremendous electrical energy and necessitates a more significant number of interconnections in the circuit.^[^ ^2^, ^3^ ^]^

Herein, we proposed a retinomorphic hardware that integrates ferroelectricity and photosensitivity within an α‐In_2_Se_3_ material, enabling simultaneous perceptive light‐sensing, memory, and computation. The devices are functionalized as photoreceptors and amacrine cells in the human retina. Besides, the coexistence of bound and mobile charges in α‐In_2_Se_3_ allows volatile resistance switching triggered by electrical and optical stimuli, and the optoelectronic memory exhibits negligible cycle‐to‐cycle variability for 10^4^ programming/erasing cycles. Additionally, the gate‐tunable out‐of‐plane (OOP) polarization dipoles in α‐In_2_Se_3_ lead to anomalous behaviors in photo‐induced short‐term plasticity. By taking advantage of dynamic response to temporal signals, the α‐In_2_Se_3_ retinomorphic sensor array can encode the images in the Mixed National Institute of Standards and Technology (MNIST) dataset for a simple readout network with a successful classification rate of ≈94% for 12 000 observations. We fabricated a 3 × 3 network of α‐In_2_Se_3_ phototransistors to conduct in‐sensor computing. The photoresponsivity (*R*) of the device can be modulated by nonvolatile polarization switching using preset voltages, allowing for the selection of five appropriate convolutional filters to convolve input information.

## 2 Results and Discussion

**Figure 1:**
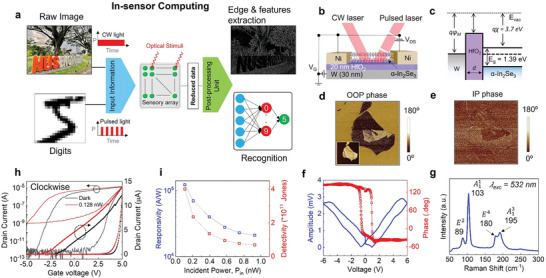
a) Computation within sensory array diagram. The information transfers to the sensory device under two forms of optical stimulation: constant wave (CW) and pulsed light. Due to the nature of synaptic behavior and gate‐controlled photocurrent in physical sensors, the data is computed within the sensors for post‐processing. b) Schematic of the device structure. c) Energy band diagram of W/HfO_2_/α‐In_2_Se_3_ transistor in equilibrium. d) Out‐of‐plane and e) In‐plane phase images of Piezo‐response Force Microscope (PFM) measurement. f) Amplitude and Phase hysteresis loop and g) Raman spectrum of ferroelectric α‐In_2_Se_3_. h) Transfer curves of α‐In_2_Se_3_ field‐effect transistor (FET) under dark and illumination conditions. i) Extracted responsivity and detectivity versus incident power, P_in_.

**Figure** **1**a outlines a sensory array data flow diagram from low‐level to high‐level processing. Image processing is associated with noise suppression, feature extraction, and edge enhancement. The recognition generally relies on the synaptic behavior of sensory devices concerning stimulus numbers to reduce data for the external readout network. Pulsed and constant wave (CW) lasers are projected onto the α‐In_2_Se_3_ phototransistor in Figure 1b to mimic information input. Concurrently, the photo‐responsivity of each pixel is electrostatically adjusted (trained) by the W gate via 20 nm HfO_2_ to output computed data. Figure S1 (Supporting Information) presents two forms of mini array (2D, 3 × 3 and 1D, 20 × 1) hardware fabricated by a common transfer technique for 2D materials in Supporting Information. The energy band diagram of the phototransistor in Figure 1c shows an electron affinity of 3.7 eV and a bandgap of 1.39 eV for α‐In_2_Se_3,_ which is compatible with visible laser wavelengths. To verify ferroelectricity in the α phase of In_2_Se_3_, we performed piezo‐response force microscope (PFM) measurement. Unintentional ferroelectric domains randomly located on the surface of 2D In_2_Se_3_ flake rendered in both out‐of‐plane (OOP) and in‐plane (IP) phase images in Figure 1d,e. The polarization domains constituted by displacement of In and Se atoms in the rhombohedral unit cell are independently distributed from surface topography (see the inset of Figure 1d). The d.c. voltage sweep from ‐6 to 6 V is applied to the conductive cantilever to examine the phase and amplitude hysteresis transition in α‐In_2_Se_3_, showing typical coercive voltages of 1.1 and −1 V as compared to reported values in the literature.^[^ ^18^ ^]^ The Raman scattering spectrum of an α‐In_2_Se_3_ flake is shown in Figure 1g, exhibiting fingerprint‐like peaks of α (*R3m*) phase and at vibration modes: *E* ^2^ (89 cm^−1^), $A_{1}^{1}$ (103 cm^−1^), *E* ^4^ (179.8 cm^−1^), $\;A_{1}^{3}$ (195 cm^−1^).^[^ ^19^, ^20^ ^]^ Transfer curves in Figure 1h show natural *n*‐type semiconductor behaviors under dark and illuminated conditions. The clockwise hysteresis loop is attributed to the polarization switching mechanism in the ferroelectric semiconductor transistor's high equivalent oxide thickness (EOT) regime.^[^ ^20^ ^]^ The α‐In_2_Se_3_ shows an excellent photo‐detection ability with considerable responsivity, *R*, and detectivity, D* of 1.2 × 10^5^ A W^−1^ and 4.02 × 10^11^ Jones, respectively, at an incident power, *P*_in_ = 0.19 nW. Extracted *R* and D* values are shown to exponentially decline versus increasing *P*_in_ in Figure 1i, which refers to the photo‐gating effect in 2D materials.^[^ ^21^ ^]^

### 2.1 Coupled Ferroelectric‐Photonic Memory with α‐In_2_Se_3_

**Figure 2:**
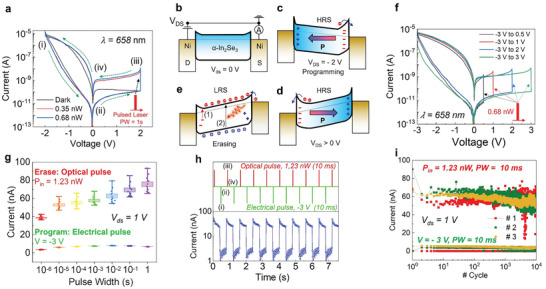
a) Dual‐swept output curves of α‐In_2_Se_3_ diode without back gate voltage. Four regimes in cycles of electrical programming and optical erasing. b–e) Sketched band diagrams correspond to various V_DS_ and illumination involved in P/E cycles. f) Hysteresis I_D_‐V_DS_ characteristics of α‐In_2_Se_3_ devices at various V_D_ sweep ranges. g) LRS/HRS ratio increases with the increase of programming width from 1 µs to 1 s. h) Pulse‐switching characteristics: using an electrical spike (−3 V, 10 ms) to program the device to HRS, and the erasing is initiated by a laser spike (1.23 nW, 10 ms). i) Endurance of α‐In_2_Se_3_ optical memory showing non‐degradation of two conductance states after 10^4^ P/E cycles.

**Figure** **2**a shows hysteresis current–voltage (I_DS_–V_DS_) characteristics of α‐In_2_Se_3_ opto‐ferroelectric memory with sweeping *V*_DS_ voltage of −2 V → 2 V → −2 V. The current sweep can be differentiated into four stages: i) electrical program, ii) read, iii) optical erase, and iv) read. The high work‐function Ni and α‐In_2_Se_3_ channel form a back‐to‐back metal–semiconductor–metal Schottky diode with a reversely biased barrier in Figure 2b.^[^ ^22^ ^]^ The forward sweep starting from −2 V, above the coercive voltage of α‐In_2_Se_3_, leads to a polarization switching between drain and source electrodes. However, these interfaces positive (negative) bound charges accumulate electron (hole), resulting in a thinner (thicker) barrier width, as shown in Figure 2c. Thus, the device operates at high resistance states (HRS, (i) electrical programming). When a positive *V*_DS_ is applied to the device (Figure 2d), the polarization of dipole moments reversely flips to the opposite direction. Consequently, a larger Schottky barrier (SB) rises in the drain electrode, which is predominantly dominated by free positive charges (hole). Therefore, resistive switching observed in a reverse sweep exhibits a negligible programming/erasing ratio in dark conditions.

When the electron and hole are confined at the Ni/α‐In_2_Se_3_ interface, a 658 nm pulsed laser (*P*_in_ = 0.35 and 0.68 nW) illuminates the device for 1 s between forward and reverse sweeps. The supplementary energy introduced by laser simultaneously performs two processes: 1) exciting the confined electron (hole) at the interface to the conduction (valence) band to reduce the SB and 2) generating electron‐hole pair to enhance channel conductance due to native photoconductivity, as depicted in Figure 2e. After the laser pulsing process, the SB solidly becomes lower due to process 1), leading to an increase of drain current in the α‐In_2_Se_3_ channel from 10^−11^ to ≈10^−8^ A, which infers that the device is effectively switched to low resistance state (LRS, iii) optical erase) (see Figure 2a). To prove that our light‐induced resistive switching distinguishes itself from the electrical reset mechanism, we carry out multiple sweeping range measurements in Figure 2f. Herein the *I*_DS_–*V*_DS_ characterizations begin with a programming voltage of −3 V, and the optical erasing voltage varies from 0.5 to 3 V. We found that all output curves share a common behavior despite the amplitude of erasing voltage, which can be above (2 and 3 V) or below (0.5 and 1 V) the coercive voltage of α‐In_2_Se_3_. In addition, Figure 2g illustrates the speed‐dependent programming (−3 V) and erasing (Pin = 1.23 nW) ratio observed in opto‐ferroelectric memory. Remarkably, our devices demonstrate rapid activation in response to exceptionally brief optical or electrical pulses, lasting merely 1 µs. This swift operation, facilitating efficient erasing and programming, is on par with conventional electrical memory technologies.^[^ ^23^ ^]^ Notably, our approach significantly mitigates energy dissipation when compared to trap‐based optical memory systems implemented in MoS_2_ heterostructures, which operate with pulse widths (PW) in the range of 0.01 s and incident power levels of 2 nW,^[^ ^24^ ^]^ or MoO_x_ optical resistive memory devices, operating with pulse widths of 0.6 s and power of 150 mW.^[^ ^7^ ^]^ Furthermore, our opto‐ferroelectric memory exhibits a low‐resistance state (LRS) to high‐resistance state (HRS) switching ratio of 40 at a pulse width of 1 µs (as depicted in Figure 2g), which increases to ≈80 as the pulse/erasure width is extended to 1 s. Importantly, the preservation of fading dynamics within the sub‐millisecond regime, as evidenced in Figure S4c (Supporting Information), serves as a distinctive benchmark for short‐term memory‐based computing.

The opto‐ferroelectric memory is a cyclically repeated program/read/erase/read sequence (PW = 10 ms) for several cycles in Figure 2h, delivering a constant on‐off ratio of 50 with the reading pulse of 1 V, 500 ms. Figure 2i shows a reliable endurance with minor cycle‐to‐cycle variation for LRS and HRS over 10^4^ cycles. In particular, we have expanded the endurance test to three distinct devices, randomly chosen from an array depicted in Figure 2i. This expansion aims to demonstrate the insignificance of device‐to‐device variation. Additionally, we regard the summarized on‐off ratio and memory windows of nine devices as crucial benchmarks to confirm minimal device‐to‐device variability within the [3 × 3] array, as discussed in Figure S2a,b (Supporting Information).

### 2.2 Gate‐Controlled Short‐Term Plasticity (STP) in Retinomorphic Sensors

**Figure 3:**
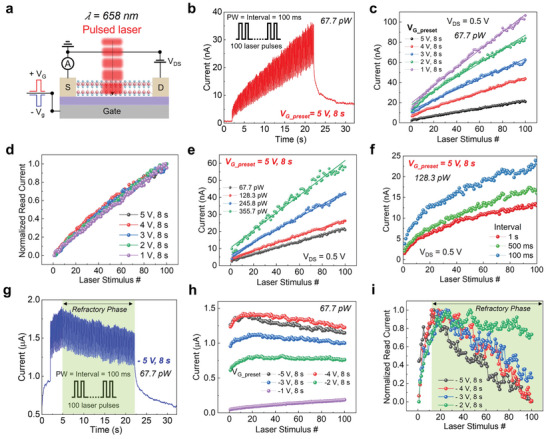
a) Measurement schematic of α‐In_2_Se_3_ optical memory, a series of optical pulses is projected on the device while an electrical pulse initially controls the polarization status. Drain current showing two opposed integration directions for 100 identical laser pulses after pre‐set by electrical pulses of b) 5 V and h) −5V for 8 s. Extracted read current at off‐laser states with various c) positive and g) negative pre‐set voltages, V_G_preset._ d) The solid lines represent the fitting results from the linear model: *y* = intercept + slope × *x* where average intercept and slope are 1.19 × 10^−8^ and 5.3 × 10^−10^ (d) and i) Normalized current versus stimulus number of (c) and (g), respectively. e) Laser‐intensity and f) frequency tunable plasticity characteristics, showing paired‐pulse facilitation (PPF).

The α‐In_2_Se_3_ optical memory can be written by optical stimulation to output power intensity‐dependent and history‐dependent conductance states, which allows us to replicate basic features of synaptic plasticity in implementing learning and memory function in the human synapse. Before being stimulated by laser pulses, various positive and negative voltages are applied to the back gate for 8 to set the channel conductance state (refer to measurement scheme in **Figure** **3**a). It is noteworthy that the amplitude and direction of the preset *V*_G_ pulse significantly impact the optical memory's fading effect. Figure S6a (Supporting Information) shows the photocurrent decay characteristics in these regimes, where a single pulsed laser pattern (*P*_in_ = 1.23 nW, 100 ms) illuminates the device to record the photocurrent dynamic. The Photocurrent fading within 10 s after stimulation can be well‐predicted by the exponential decay function: $I=I_{0}+A_{1}e^{-\left(t-t_{0}\right)/t_{1}}+A_{2}e^{-\left(t-t_{0}\right)/t_{2}}$, where time constants *t*_1_ and *t*_2_ imply the fast and slow response. The summarized *t*_1_ and *t*_2_ varying with respect to preset *V*_G_ amplitude in Figure S6b (Supporting Information) shows two prominent regimes of decay time when *V*_G_ shifts from negative to positive. The fast decay time, *t*_1_, varies ≈60 ms with a positive *V*_G_preset_. However, when the preset voltage sweeps to higher negative values, it doubles to ≈120 ms. Besides, the slow decay time, *t*_2_, which originates from the shallow trap density in α‐In_2_Se_3_, exhibits a similar behavior with an increasing range of ≈1–3.2 s.^[^ ^21^ ^]^ This quantitative analysis of time decay reveals that the appropriate gate voltages are applicable to modulate the fading effect in the optical memory, leading to unambiguous variations of temporal dynamics with respect to *V*_G_preset_. Due to photon‐triggered short‐term plasticity, the α‐In_2_Se_3_ device shows two opposing light‐induced dynamic responses. When the positive preset voltage is applied, the pristine ferroelectricity in the channel forms a dipole polarization, causing upward band bending at α‐In_2_Se_3_/HfO_2_ interface. The elimination of mobile charges in the channel creates a high‐resistance path. The energy band diagram sketched in Figure S3 (Supporting Information) *a* and *b* indicates that photon energy given by optical pulse simultaneously excites the bound charge in the valence band to increase channel conductivity and release the local band bending. The Photocurrent is accumulated under frequent optical stimulation (e.g., 100 laser stimuli with PW = interval = 100 ms, *P*_in_ = 67.7 pW), showing a perfect linear integration after 100 identical laser stimuli, as shown in Figure 3b. The devices are therefore functioning as graded neurons. Although the linearity of optical facilitations stays unchanged, Photocurrent increases with different degrees of slopes, corresponding to different positive *V*_G_preset_ (e.g., linear fitting line in Figure 3c,d). This issue can be understood as different resistive switching levels induced by the initial *V*_G_preset._ It is noted that every post‐synapse current value is read under laser‐off conditions immediately after the optical writing pulse (see Figure S6c, Supporting Information). The linear fitting solid lines in Figure 3c show an average *R* ^2^ value of 0.99242 over five *V*_G_preset_ amplitudes.

Furthermore, the device conductance mimics synaptic strength in biological systems, persistently increased concerning light‐dosage intensity illuminated on volatile α‐In_2_Se_3_ optical memory, as shown in Figure 3e. The fitting results preserve satisfactory linearity when the overall incident power increases with an average *R* ^2^ = 0.99145. We examine paired‐pulse facilitation (PPF) in Figure 3f with different frequencies of optical stimulation, demonstrating interval‐dependent conductivity. In which the variation of PPF ratio ((*A*_2_‐*A*_1_)/*A*_1_) shows an exponential decay following the double decay function: PPF = C_0_ + *C*_1_e^−Δt/2^ +*C*_2_e^−Δt/2^ (Figure S4, Supporting Information).

Conversely, the light‐induced plasticity exhibits a photocurrent anomalously dependent on the number of stimuli under the negative preset voltage regime. In this regard, we characterized the retinomorphic sensor under the same illumination power and *V*_G_preset_ set to −5 V. Interestingly, the facilitation of Photocurrent is pronounced only at the first 20 optical pulses (PW = interval = 100 ms; Figure 3g) and reaches maximal facilitation (firing), followed by saturation and significant depression for subsequent 80 stimulation pauses. Two distinct periods can be identified in the behavior of the spiking neuron: integration and refractory phases. In this context, the spiking state might refer to the initial weight values assigned to the network's neurons, often set to small random values. After entering the refractory phase, the neuron becomes unresponsive to optical stimulation, thereby restricting the transmission of information through the neurons.^[^ ^25^, ^26^ ^]^ With negative *V*_G_preset_, the dipole moment in the α‐In_2_Se_3_ channel experiences downward polarization, leading to a low‐resistance state (LRS) due to an accumulation of free carriers at the HfO_2_/α‐In_2_Se_3_ interface, as shown in Figure S3c,d (Supporting Information).^[^ ^20^, ^23^ ^]^ This results in initially high conductivity, providing fast facilitation at early stimulation pauses due to photogenerated carriers. However, the photon energy spontaneously excites bound charges in polarized dipoles to the conduction and valence bands, implying a polarization loss. Therefore, the interfacial band bending is released, leading to a decrease in overall photoconductivity. Consequently, after about 20 optical stimuli pulses, the facilitation reaches its maximum value and decreases, despite unchanged stimulation conditions. The off‐field read current versus stimulus number curves depicted in Figure 3h show that the depression after maximal facilitation only occurs when the absolute value of negative *V*_G_preset_ is higher than the coercive voltage of α‐In_2_Se_3_ (−1 V). In plots of normalized read current, it can be seen in Figure 3i that maximal facilitation is shifting to an earlier stimulus number while the degree of depression tends to be more intensive when negative *V*_G_preset_ amplitude increases.

### 2.3 Highly Accurate Hand‐Written Digits Classification

**Figure 4:**
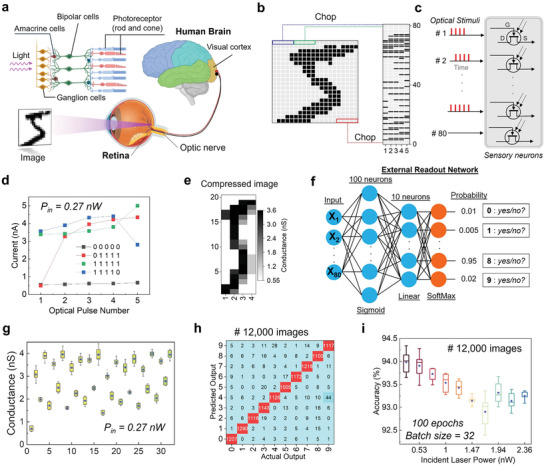
a) Human visual system diagram describing the biological retina within the eyeball that connects to the visual cortex in the human brain via an optical nerve. b) 1‐bit depth grayscale image [20 × 20] in the MNIST dataset was chopped sequentially into 80 [5 × 1] rows, which then converted to c) 80 temporal laser stimuli. d) Response currents of α‐In_2_Se_3_ optical memory after being streamed by four different optical pulse sequences confirm the fading effect in short‐term memory. e) Conductance matrix representing the compressed MNIST image after pre‐processing within the sensor. f) A simple neural network performs the perception section in the visual system with fully connected neurons, probability of classification results is computed via Soft‐max regression. g) The final conductance states 32 identical pulse streams containing only bits ‘0’ and ‘1’ in a chain of 5 pixels. h) Confusion matrix representing classification results for 12 000 images in the testing phase. i) The dependence of recognition accuracy on incident power, P_in_ of optical stimuli.

The biological human retina is the innermost eye layer responsible for capturing and processing visual information. It consists of several distinct types of cells (e.g., photoreceptor cells, bipolar cells, ganglion cells, and amacrine cells) that collaborate to create a perception of the world around us.^[^ ^27^ ^]^ To simulate the biometric retina, the retinomorphic hardware in this work partly mimics the photoreceptor cells, which are responsible for detecting light, and the amacrine cells, which encode the data using short‐term plasticity features within a sensory device (**Figure** **4**a). A 1‐bit depth grayscale 20 × 20 image in the MNIST dataset is divided into groups of 5 pixels, starting from the top left and moving to the bottom right, forming 80 [5 × 1] matrices, as shown in Figure 4b. The black and gray pixels for bits ‘0′ and ‘1′ in each row are then converted to an optical temporal signal (e.g., PW = 100 ms, interval = 500 ms). As discussed in the above section, the temporal dynamic behavior in the α‐In_2_Se_3_ sensor enables different photoconductivity states for different inputs. Figure 4d presents four examples of response photocurrent of four optical stimulations. Figure S7b (Supporting Information) illustrates the electrical waveforms corresponding to the output of remote‐sense and switch unit (RSU) channel 1, while the optical pulse represents the chain 01100 pulse stream in RSU channel 2. Figure S7c (Supporting Information) exhibits real‐time current readings for four distinct temporal patterns: 01100, 10101, 00101, and 10101, across ten different incident power levels, *P*_in_. All temporal responses reveal sequential conductance levels proportional to increasing *P*_in_ values, indicating that the data is unaffected by circuit noise, thus facilitating further software readout function. The real‐time measurement scheme for several pulsed laser sequences in Figure S7c (Supporting Information) leverages excellent facilitating linearity and historical capture ability of α‐In_2_Se_3_ to generate distinct conductance states.

A chain of 5 pixels composed of two bits (0 and 1) results in a maximum of 2^5^ = 32 different arrangement cases. Therefore, we measured 32 identical final conductance states corresponding to 32 optical inputs streamed through the device (Figure 4g). These conductance values reconstruct the digit image in Figure 4e, indicating compressed data precomputed within the sensor. We feed flattened conductance matrices into the readout network as the input layer for the training and testing phase. It is noteworthy that two hidden layers construct the neural network; the input data transmits forward through 100 neurons activated by the sigmoid function and ten neurons of a linear function. The SoftMax regression function calculates the probability for ten output neurons to determine the possible decision, as shown in Figure 4f. After training 48 000 images of hand‐written digits for 100 epochs and batch size of 32, the fully connected weight matrix of 2 hidden layers in a neural network form. We obtained a remarkable classification result of ≈94% when carrying out a testing phase with the joint of the rest 12 000 images. Figure 4h displays the detailed confusion matrix. To further explore the effect of photogating on training/testing precision, we incorporate tunable laser intensity of optical pulse in the programming process. Figure 4i exhibits a difference in testing accuracy with respect to incident power, where the accuracy gradually decreases with increasing *P*_in,_ and eventually, it saturates at 93% when *P*_in_ exceeds 1.47 nW. The accuracy variation with *P*_in_ is attributed to in influence of the photogating effect on responsivity, discussed in Figure 1i. Remarkably, when directly subjected to the external readout network, the MNIST data exhibited a mere 10% accuracy during the testing phase, consistently persisting at this low level throughout 100 epochs. In contrast, once encoded through the dynamic response of α‐In_2_Se_3_ graded neurons, the original data demonstrated a noteworthy accuracy rate of ≈95%. This accuracy level closely approximates the ideal value of 98% achieved by utilizing a software‐based conventional convolutional neural network (CNN) (see Figure S8b, Supporting Information).

### 2.4 Convolution Image Processing with α‐In_2_Se_3_ Phototransistor

**Figure 5:**
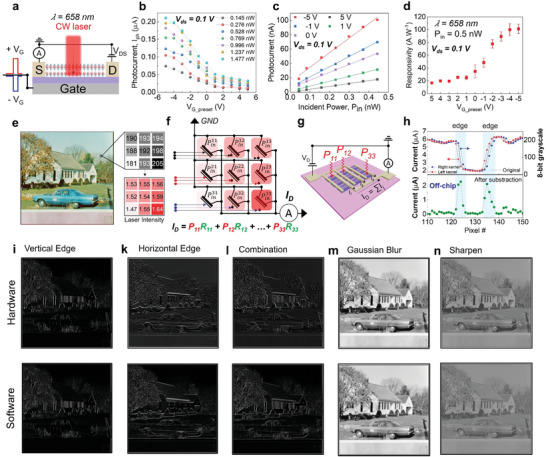
a) Measurement scheme for the optical sensor, we used an initial V_G_ pulse to train the device before sensing the CW laser. b) Variation of photocurrent with respect to initial voltage pulse amplitudes under increasing P_in_. c) The linear dependence of photocurrent on photon energy. d) The fitting slopes are functioned by initial *V*_G_ pulse amplitude, extracting photo‐responsivity in the α‐In_2_Se_3_ image sensor. e) The 8‐bit grayscale original image is converted analog signal of laser intensity for projection on a 3 × 3 optical kernel. f) Electrical configuration and g) measurement scheme of 3 × 3 array. The nine transistors in the array are individually trained by nine different back gates while sharing one drain/source terminal. h) Principle of edge enhancement after a simple arithmetic calculation. Image processing results after computed by our optical kernel and software i) Vertical edge, k) Horizontal edge, l) Combination, m) Gaussian Blur, and n) Sharpen.

Expanding from diverse computational tasks of gate‐tunable photo‐response in α‐In_2_Se_3_ retinomorphic sensor, we process the convolution operation for a 512 × 512 image within the sensory array (**Figure** **5**e, from USC‐SIPI database).^[^ ^28^ ^]^ Unlike the abovementioned temporal dynamic characteristic, the constant wave (CW) laser in this task expresses the input data while the a.c gate voltages (PW = 8 s, refer to the schematic in Figure 5a) independently preset the resistance states in the channel. Figure 5b,c exhibits light dosage‐dependent and *V*_G_preset_ amplitude‐dependent photocurrent, *I*_ph_, respectively. In the in‐sensor computing paradigm, the sensory device couples with feature extraction circuits in a single physical hardware, where the responsivity is electrostatically adjusted to obtain the desired output. It is important to note that the ferroelectric‐induced memory effect in the α‐In_2_Se_3_ channel allows us to switch the resistance state once at the beginning before light sensing. This approach is a more efficient strategy for reducing energy consumption than continuously applying electrical voltage during the convolution operation of electrostatic ambipolar photodiodes.^[^ ^2^, ^3^ ^]^ The output photocurrent (*I*_ph_) magnitude exhibits a linear relationship with the stimulus intensity, where the amplitude and direction of *V*_G_preset_ give a signification variation for linear fitting slopes of *I*_ph_ versus *P*_in_, as shown in Figure 5c. Similar to the previously mentioned gate‐tunable conductivity, photo‐responsivity (*R*) extracted from the fitting slope is proportional to the conductance levels of the channel. The multiple states of identical *R*‐value, as summarized in Figure 5d, indicate that each phototransistor can be a self‐governing pixel in the kernel of an optical filter.

We configure a 3 × 3 crossbar array of α‐In_2_Se_3_ phototransistors (see circuit diagram in Figure 5f), in which all pixels share a drain/source electrode for collecting total Photocurrent while the gate electrodes are separately controlled. Each device within our [3 × 3] array demonstrates a substantial on‐off ratio of ≈10^7^ and an ON current of ≈1µA µm^−^¹, as evidenced in Figures S10 and S2 (Supporting Information). These results signify that the properties of devices within the array exhibit non‐degradation in field‐effect mobility and conductivity compared to standalone devices found in the literatures.^[^ ^20^, ^23^ ^]^

Each pixel in the original image (Figure 5e) in 8‐bit depth grayscale linearly reflects the analog signals of laser intensity. A 3 × 3 patch of the image is then projected onto the array of bottom‐gated phototransistors using a laser spot, which can adjust the brightness using variable optical attenuators (VOA) (refer to measurement setup schematic in Figure S9b, Supporting Information). The nonvolatile behavior of the dipolar ferroelectric domain in channel α‐In_2_Se_3_ allows us to initially device‐by‐device program all nine pixels by nine back‐gate electrodes via source measurement unit (SMU) in the analyzer before optical stimulation. The voltage values (amplitude and direction) in circuit configuration, e.g., *V*_11_, *V*_12_, …, and *V*_33_ (Figure S9c, Supporting Information), are predesigned to replicate specific kernels stored in *R* (responsivity) values of each device for different processing purposes.

The optical image of the phototransistor array and their transfer curves are presented in Figures S9a and S10 (Supporting Information), respectively. As referred to gate‐tunable *R*‐values in Figure 5d, a 3 × 3 matrix of *V*_G_preset_ is applied to the array to set an appropriate 3 × 3 photoresponsivity (*R*) matrix. For edge enhancement, we use the kernel matrix stored in *R* to replicate the Prewitt filter in typical software‐based convolution image processing, e.g., $\left[\begin{array}{ccc} 5\;V & -1\;V & -5\;V\\ 5\;V & -1\;V & -5\;V\\ 5\;V & -1\;V & -5\;V \end{array}\right]$ and $\left[\begin{array}{ccc} -5\;V & -1\;V & 5\;V\\ -5\;V & -1\;V & 5\;V\\ -5\;V & -1\;V & 5\;V \end{array}\right]$ for the left and right vertical edges, respectively. The detail 3 × 3 matrices of *R* correspond to these 3 × 3 *V*_G__preset in Figure S11 (Supporting Information), indicating the construction of five convolutional kernels based on responsivity matrices. The subtraction of these matrices in a differential pair represents kernels' negative and positive sides. The output photocurrent, ***I*_ph_**, corresponds to the multiply‐accumulate operation of responsivity matrix ***R*** and local power ***P*_in_** of the incident laser as follows ***I*_ph_** = ***R*** × ***P*_in_**.^[^ ^2^, ^11^ ^]^ Photocurrent in the *j*th column is achieved by *I_j_* = ∑ *R_ij_* × *P_ij,_* and the summation of total current can be implemented by the crossbar array using Kirchhoff's law. After sliding the 3 × 3 patch over the image, the simultaneous accumulation generates a 510 × 510 photocurrent matrix. In the post‐processing unit, a simple absolute subtraction of the left and right ***I*_ph_** matrices results in a significant enhancement of edge pixels relative to the background pixels, as shown in Figure 5h. Figure S12 (Supporting Information) is an example of the overall process with a solid edge extracted from the original image. The convoluted images of vertical and horizontal edges displayed in Figure 5i,k, respectively, show satisfactory precision compared to software‐based convolutions.

In particular, the device‐to‐device variation is a critical factor influencing the effectiveness of background subtraction in the convolutional process. Therefore, two phototransistor arrays with different degrees of device‐to‐device variability are applied to convolute the image in Figure S13 (Supporting Information). It is evident that the sensor, which possesses slight pixel variation in responsivity *R*, reveals an effective edge enhancement relative to the background. In contrast, the solid device‐to‐device variation array may not provide the symmetry *R* values in the negative and positive sides of the kernel after subtraction to represent the Prewitt filter. By tracking the pixel‐to‐pixel difference of parallel images from two sources (hardware and software), we found that the structure similarity scores (SSS) of vertical, horizontal edges and combined images were 87%, 70.5%, and 77.35%, respectively, the comparison detailed in Figure S14a (Supporting Information). In addition, the application of our hardware‐based convolution approach expands to a Gaussian filter for efficient blurring and sharpening, while the Difference of Gaussian (DoG) (Figure S14b, Supporting Information) kernel is a feature enhancement algorithm.

## 3 Conclusion

In summary, we have demonstrated a novel retina‐inspired visual sensor that utilizes 2D ferroelectric α‐In_2_Se_3_ for in‐sensor computing. This approach has functionally incorporated two features in semiconducting α‐In_2_Se_3_ channels, excellent photo response and nonvolatile electrical polarization, to create an optoelectronic memory that enables simultaneous perceptive light‐sensing, memory, and computation. The device demonstrated short‐term plasticity in response to optical stimulation, exhibiting both facilitation and depression. We implemented retinomorphic computing in various computer vision tasks, including encoding and classifying 12 000 images of hand‐written digits into their corresponding numerical values. In addition to the electrostatic‐trained photoresponsivity, the device can perform in‐sensor convolution image processing by pre‐programming different kernels into 3 × 3 sensor networks via local back gates. These results manifest the potential of our retinomorphic sensor for a wide range of applications, including image recognition and processing, machine learning, and artificial intelligence.

## 4 Experimental Section

### Device Fabrication

The Metal and Dielectric Sputtering AJA system deposited a 30 nm tungsten (W) layer on the p^++^ Si/SiO_2_ (285 nm) substrate. The Laser Writer was carried out to pattern back gate electrodes in two forms: 3 × 3 and 20 × 1 array, as shown in Figure S1 (Supporting Information). The whole chip was then immersed in W etchant for 25 s to remove non‐covered W. The back‐gate electrodes were rinsed with organic solvents and covered by 20 nm HfO_2_ by 200 cycles of atomic layer deposition (Picosun ALD) at 150 °C for the dielectric layer. This approach facilitated the expansion of the array dimensions to a larger scale, specifically to a [5 × 5] 2D array, while maintaining a gate footprint of 5 × 7 µm^2^, as depicted in Figure S15a (Supporting Information). Furthermore, the array size was extended to a [10 × 10] configuration compatible with back‐end‐of‐line requirements. Notably, the minimum gate length achieved in this context is ≈2 µm, as illustrated in Figure S15b (Supporting Information). In the case of a 1D array configuration, the method also supported the creation of an [30 × 1] array, as shown in Figure S15c (Supporting Information).

After that, an exfoliated α‐In_2_Se_3_ flake from the commercial bulk crystal was transferred to back gate electrodes. Drain/Source electrodes are patterned by ultrahigh‐performance electron‐beam lithography (EBL) Raith EBPG5200 with Nickel (Ni) contacts deposited by ultrahigh vacuum e‐beam evaporator, AJA system at 10^−8^ Torr.

### Material and Device Characterization

The α‐(*R3m*) rhombohedral phase of In_2_Se_3_ was confirmed by Renishaw inVia Raman scattering spectroscopy dual‐laser: 532 and 325 nm. Piezo‐response and surface morphology measurements were performed by atomic force microscope (AFM) Park System NX20 with built‐in PFM function. Keysight B5100A Semiconductor Device Analyzer measured the electrical characteristics in ambient conditions. The photo response measurement setup under the illumination of a 658 nm wavelength CW laser with controller Thorlabs ADR‐1805 with SMA modulator. An in‐series connection with B1531A RSU supported the transient pulsed laser generation.

### Pulsed Laser Characterization

In specific measurement setup, the Keysight B5100A Semiconductor Device Analyzer with a high‐resolution and high‐speed ADC was utilized within the high‐power source measurement unit (HPSMU). A comprehensive experimental configuration is displayed in Figure S7a (Supporting Information), wherein dual channels of Waveform Generator Fast Measurement Units (WGFMU) were employed to generate controlled electrical pulses with precise intervals and amplitudes. At sampling intervals, current and voltage measurements were obtained at no less than 5 ns. The resulting output waveforms were subsequently directed to two remote‐sense and switch units (RSU). The waveform in RSU Channel 1 was interfaced with the laser modulatory terminal in the laser controller to administer pulsed optical stimulation. In contrast, the waveform in RSU Channel 2 was applied to the drain electrode of the field‐effect transistor (FET) for conductance readings. The high‐resolution source measurement unit (HRSMU) within SMU 1 enabled to adjust the measurement range to ≈1 pA.

The Mixed National Institute of Standards and Technology (MNIST) dataset contains training samples of 60 000 images and testing samples of 10 000 images (28 × 28 pixels) representing digits written by high school students and United States Census Bureau employees. For the recognition task in Figure 4, a supervised learning algorithm, SoftMax regression, was used to train the readout function via the Keras toolkit in Python, which provided high‐performance programming by accessing TensorFlow was used to train the readout function.

## Conflict of Interest

The authors declare no conflict of interest.

## Supporting information

Supporting Information

## Data Availability

The data that support the findings of this study are available from the corresponding author upon reasonable request.
