# Supplementary material for: Coupled Ferroelectric‐Photonic Memory in a Retinomorphic Hardware for In‐Sensor Computing
Source: Adv Sci (Weinh). 2024 Jan 17;11(12):2303447. doi: 10.1002/advs.202303447 (PMC10966535; doi:10.1002/advs.202303447)
Supplement: Supplementary file 1 — Supporting Information [file ADVS-11-2303447-s001.pdf]

ADVANCED SCIENCE
Open Access

Supporting Information

for *Adv. Sci.*, DOI 10.1002/advs.202303447

Coupled Ferroelectric-Photonic Memory in a Retinomorphic Hardware for In-Sensor Computing

*Ngoc Thanh Duong, Yufei Shi, Sifan Li, Yu-Chieh Chien, Heng Xiang, Haofei Zheng, Peiyang Li, Lingqi Li, Yangwu Wu and Kah-Wee Ang**

Supplementary Information

Coupled Ferroelectric-Photonic in a Retinomorphic Hardware for In-sensor Computing

*Ngoc Thanh Duong, Yufei Shi, Sifan Li, Yu-Chieh Chien, Heng Xiang, Haofei Zheng, Peiyang Li, Lingqi Li, Yangwu Wu, and Kah-Wee Ang**

N. T. Duong, Y. Shi, P. Li, Y-C. Chien, H. Xiang, H. Zheng, L. Li, S. Li, Y. Wu, and Prof. K-W. Ang
Department of Electrical and Computer Engineering, National University of Singapore, 4 Engineering Drive 3, Singapore, 117583 Singapore.
E-mail: eleakw@nus.edu.sg

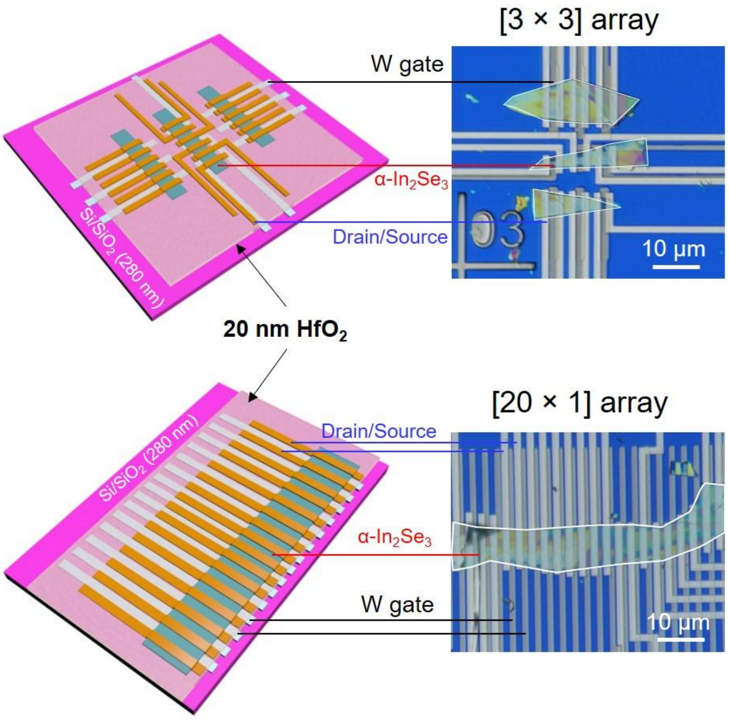

Figure S1. 3D schematic and optical images of α-In_2Se_3 device fabricated in 2D [3 × 3] and 1D [20 × 1] array scale. Metal Sputtering deposited a 30 nm tungsten (W) on p^{++} Si/SiO_2 (285 nm) substrate. A laser writer carried out the patterning process to partially protect the back-gate electrode area. The chip was then immersed in W etchant for 25 seconds to remove non-covered W. The back-gate electrodes were rinsed with organic solvents and covered by 20-nm HfO_2 by 200 cycles of atomic layer deposition (Picosun ALD) at 150º C for the dielectric layer. After that, we accurately transferred an exfoliated α-In_2Se_3 flake from the bulk crystal to the back-gate electrodes. Drain/source electrodes are patterned by Ultra-High-Performance electron-beam lithography (EBL) Raith EBPG5200, followed by Ultra High Vacuum E-Beam Evaporator deposits nickel (Ni) contacts with AJA system at 10^{-8} Torr.

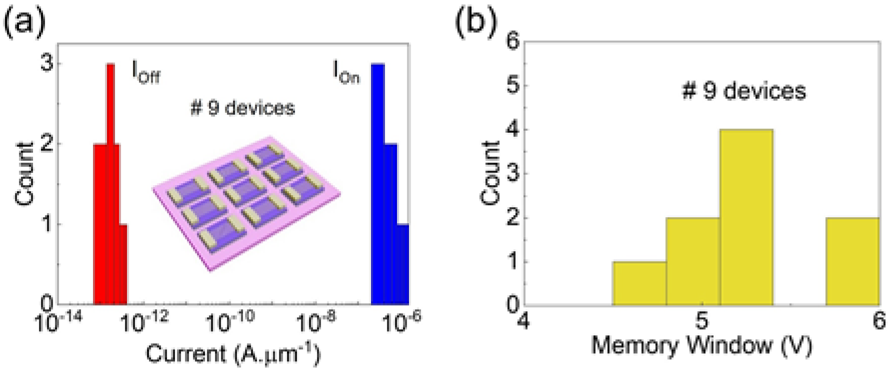

Figure S2. (a) The histogram distribution of On and Off current and (b) Memory Windows extracted from dual-sweep transfer curves of nine devices randomly selected in arrays.

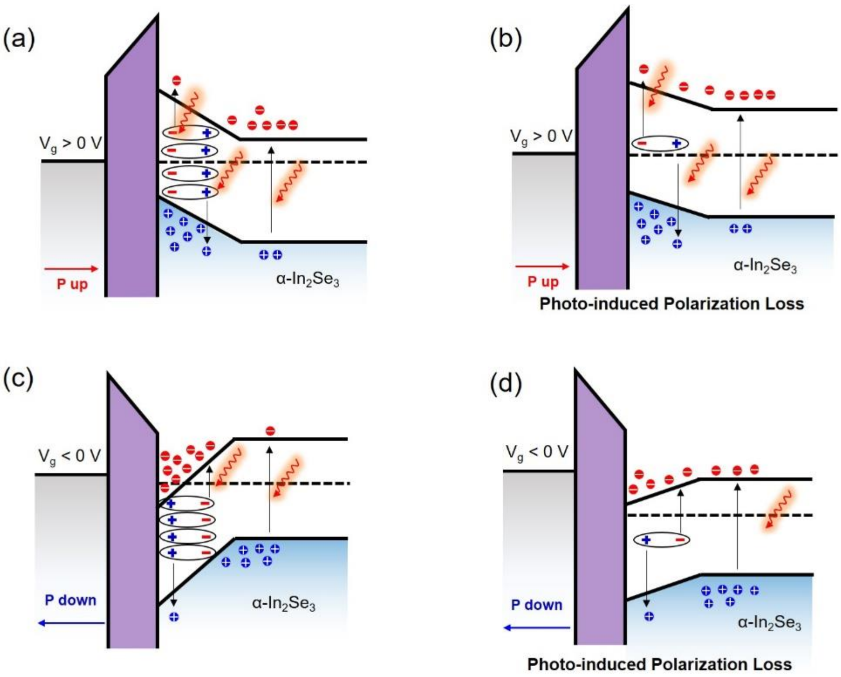

Figure S3. Energy band diagram of α-In_2Se_3 phototransistor at different V_{G_preset} schemes.

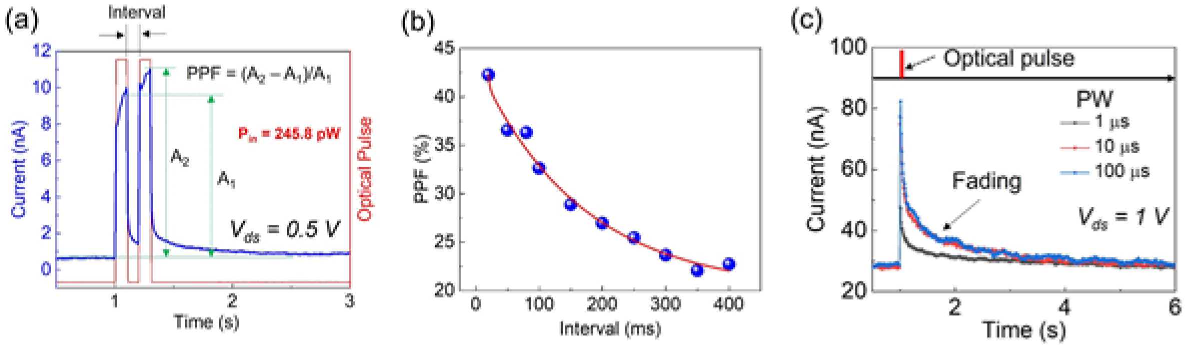

Figure S4. (a) Laser-induced paired-pulse facilitation (PPF) and definition of A_1, A_2 amplitudes, and (b) PPF ratio = $(A_2 – A_1)/A_1 \times 100$ % exponentially decays when the interval between two pulses increases. (c) Dynamic response of photocurrent with sub-millisecond pulse-width of the optical pulses, e.g., 1 µs, 10 µs and 100 µs.

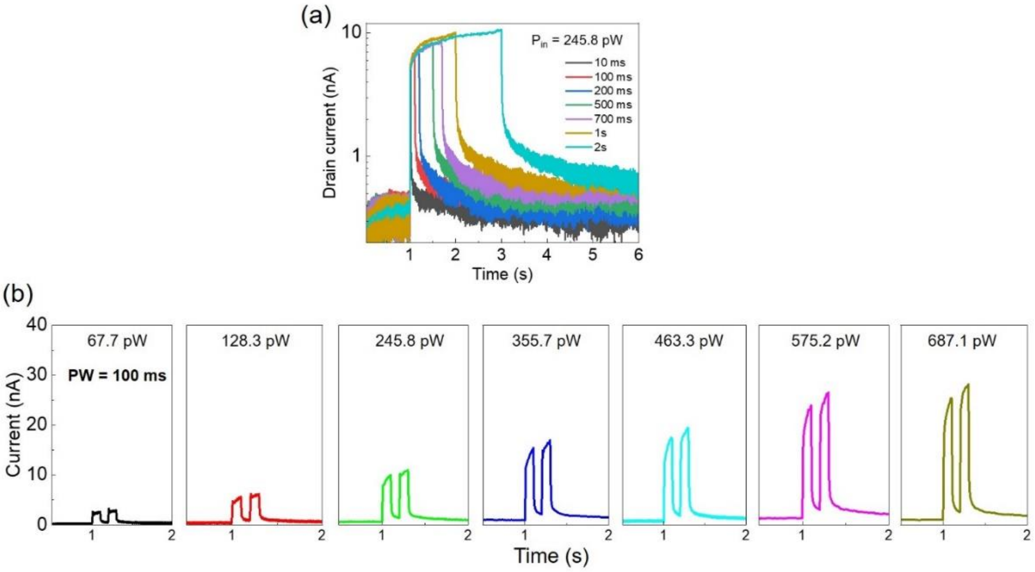

Figure S5. (a) Different dynamic responses of photocurrent with increasing PW (10 ms to 2 s) of a pulsed laser caused by trap density within HfO_2. (b) Higher photocurrent concerning increasing incident power.

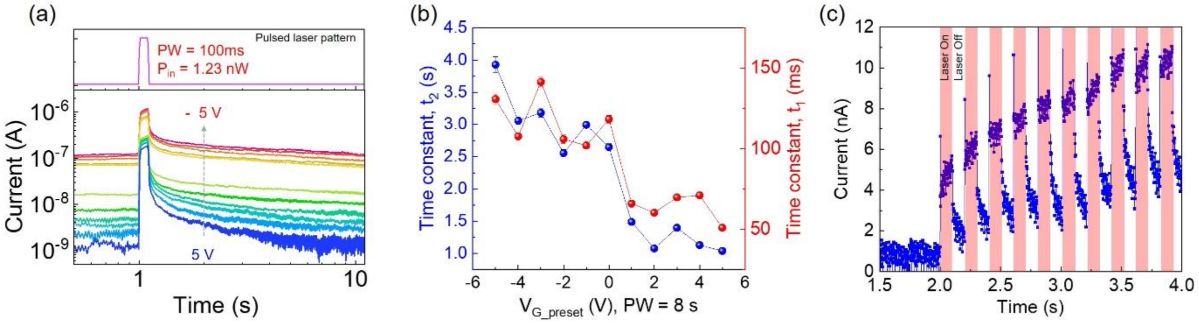

Figure S6. (a) Temporal response of α-In_2Se_3 phototransistor to a single pulsed laser pattern (100 ms, 1.23 nW) under various V_{G_preset} pulses (-5 V to 5 V, step 1 V, for 8 s). (b) Extracted retention time from exponential decay functions, exhibiting two difference mechanism. (c) Raw photocurrent data present integration of measured after several laser stimuli.

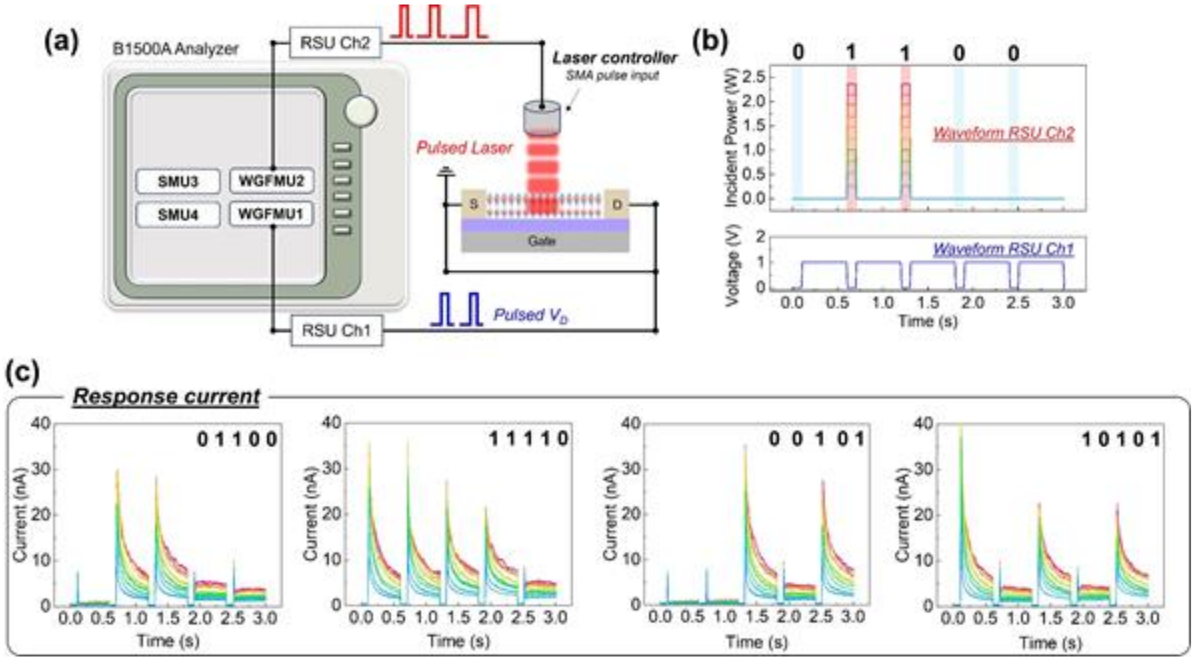

Figure S7. (a) Measurement setup for pulsed laser controller and voltage using B1500A Semiconductor Device Analyzer (b) Output waveform channel 1 and channel 2 of RSU and temporal dynamic of fading α-In_2Se_3 optical memory concerning discrete pulse streams. (c) Real-time measurement of four different temporals consisting of bit '0' and '1': 01100, 10101, 11110, and 00101. The bit '1' is represented by a laser pulse (PW = 100 ms, P_{in} = 0.27 nW to 2.36 nW (step 0.27 nW).

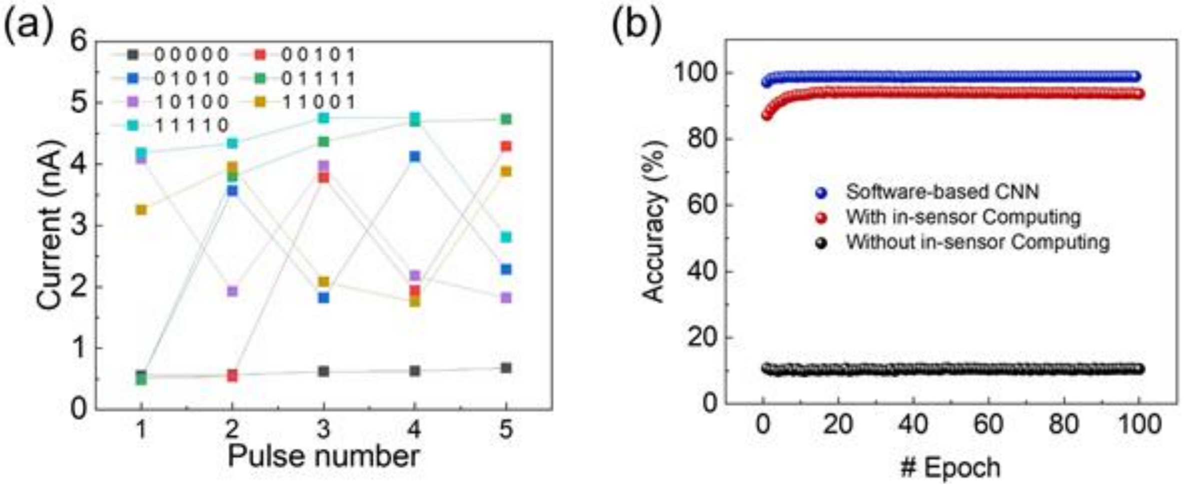

Figure S8. (a) The summary response current of seven different optical pulse sequences indicated seven distinct final conductance levels. (b) Comparison of accuracy progress in testing phases over 100 training epochs in three models: software-based convolution neural networks (CNN), with and without our in-sensor encoded data using α-In_2Se_3 optoelectronic memory.

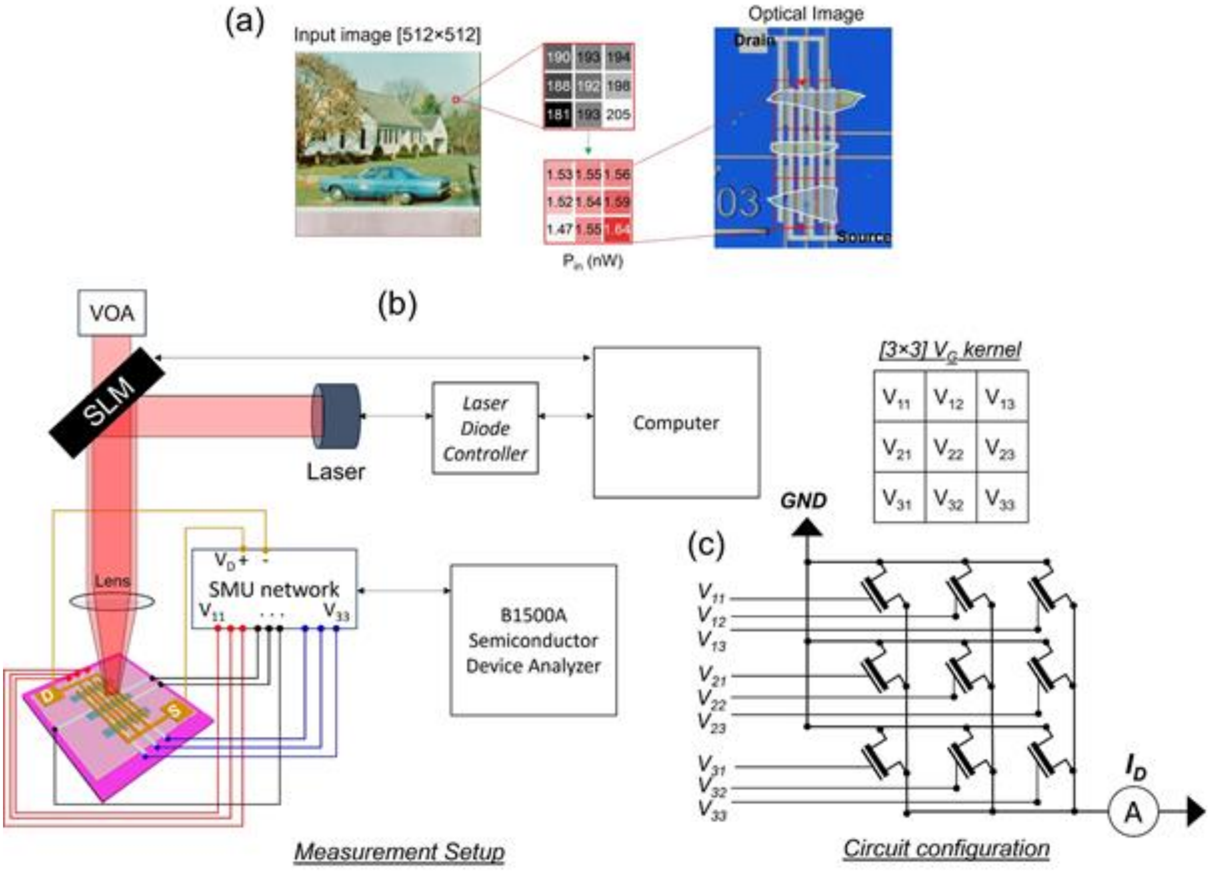

Figure S9. (a) Optical image and its schematic of the 3×3 phototransistor crossbar array used in convolution image processing. (b) Hardware setup for in-sensor computing characterization. (c) The circuit configuration of [3×3] α-In_2Se_3 phototransistor array, implementing convolution image processing. The [3×3] α-In_2Se_3 phototransistor array is positioned on a sample holder with an x-y micromanipulator under a microscope with an objective lens (**Figure S9b**). The non-volatile behavior of the dipolar ferroelectric domain in channel α-In_2Se_3 allows us to manually device-by-device program all nine pixels by individual back-gate electrodes via source measurement unit (SMU) in the analyzer before optical stimulation. The voltage amplitude in circuit configuration, e.g., V_{11}, V_{12}, …, and V_{33} (**Figure S9c**), are pre-designed to replicate specific kernels for different processing purposes. Once electrostatically activated, all pixels within the array respond to the 658-nm wavelength laser diode, powered by a voltage controller (Thorlabs, ADR-1805) and programmatically controlled via a spatial light modulator (SLM). The objective lens precisely projects the laser beam onto the phototransistor array. The resulting photocurrent in the *j*th column, represented as I_j, is computed as the summation of the product of R_{ij} (responsivity) and P_{ij} (incident optical power) for each pixel in the column. The total current's summation is efficiently realized by applying Kirchhoff's law within a crossbar array configuration.

In the future ideal setup, the [3 × 3] phototransistor array should be wire-bonded into a 24-pin chip holder and connected to an acquisition system. [1] Therefore, the output information of on-chip computing can directly interface with a post-processing unit (off-chip) for further calculation. [2] The presented circuit configuration in **Figure S9c** relies on an array of α-In_2Se_3-

based phototransistors to perform convolution processing. In this configuration, electrical symbols denote optoelectronic devices, with their photo-response being effective to independent modulation through gate voltage control. After investigating all photoresponsivity (R) values of each pixel in the array, the resulting photocurrent at the output indicates the cumulative summation of photocurrents generated by individual devices within the hardware.

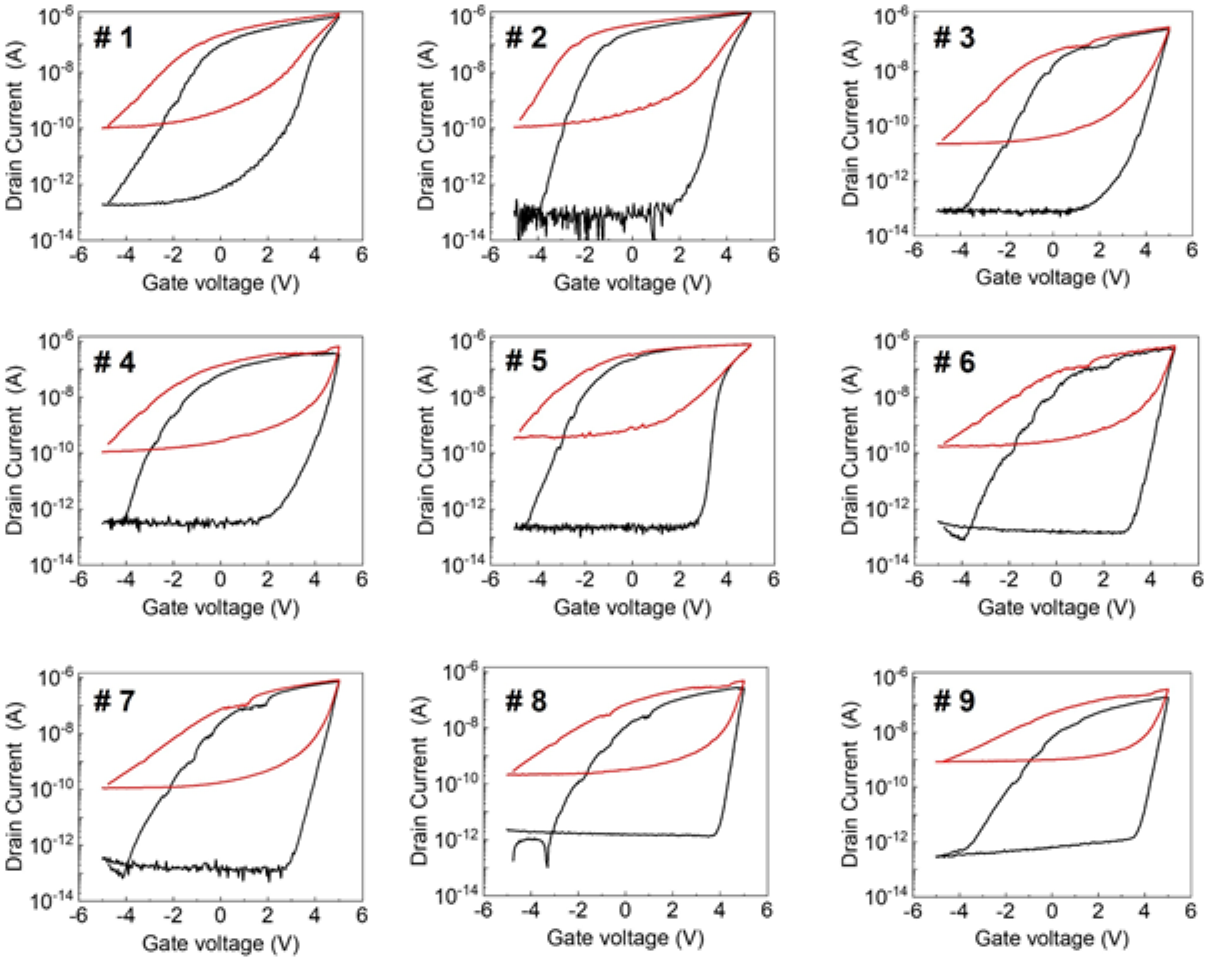

Figure S10. Transfer curves under the dark and illuminated condition of nine individual phototransistors extracted from 3 × 3 pixels. All curves show clockwise hysteresis loops.

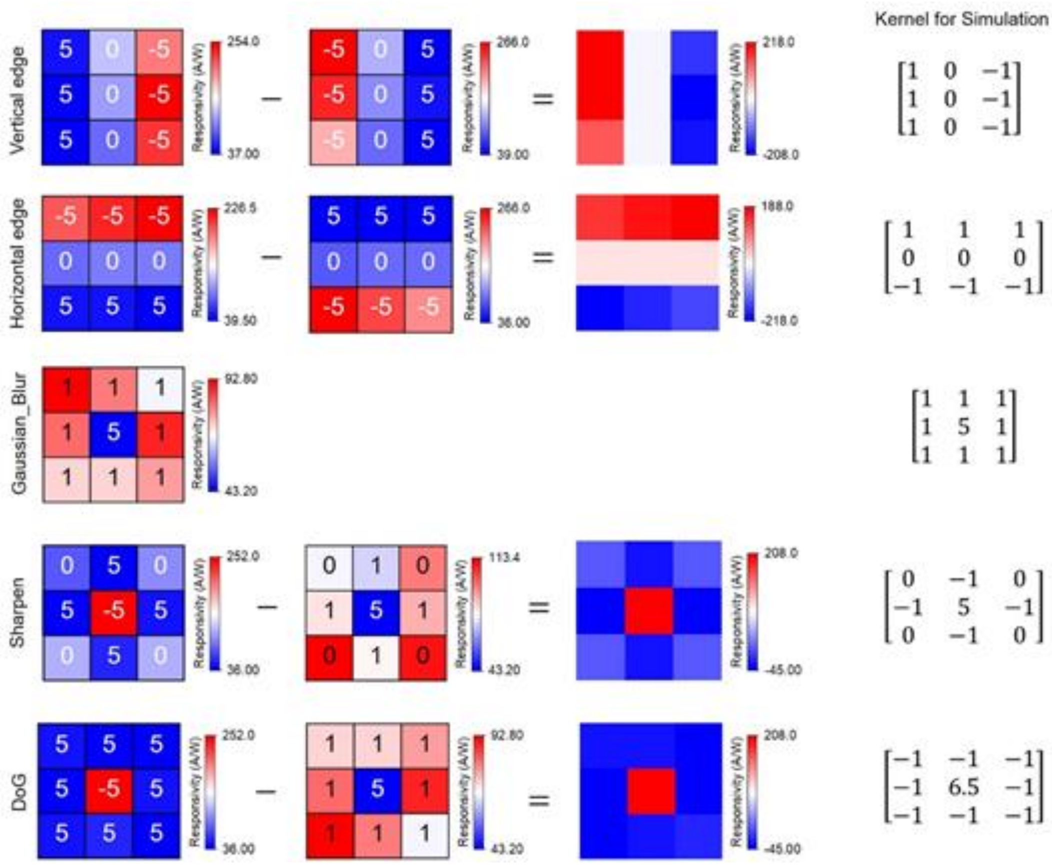

Figure S11. Programmed photoresponsivity for five convolutional kernels and their corresponding numerical kernels for software simulation. The inset numbers represent the amplitude of the preset voltages applied to the back gate, V_{G_preset}.

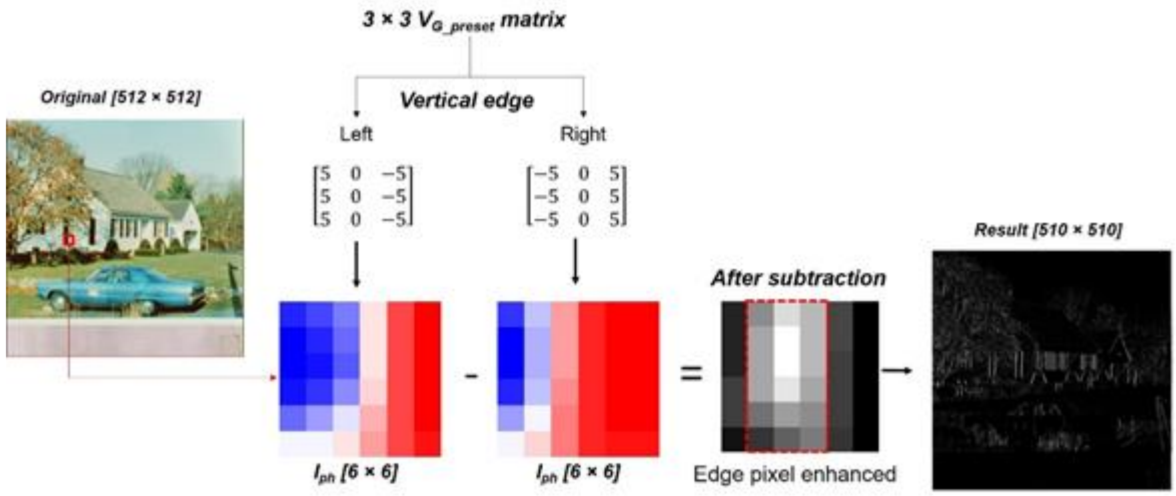

Figure S12. Process of convolution using the Left and Right kernels and software subtraction to enhance the edge pixels. We use a patch of 6×6 pixels from the original image with a solid edge as an example to visualize the process.

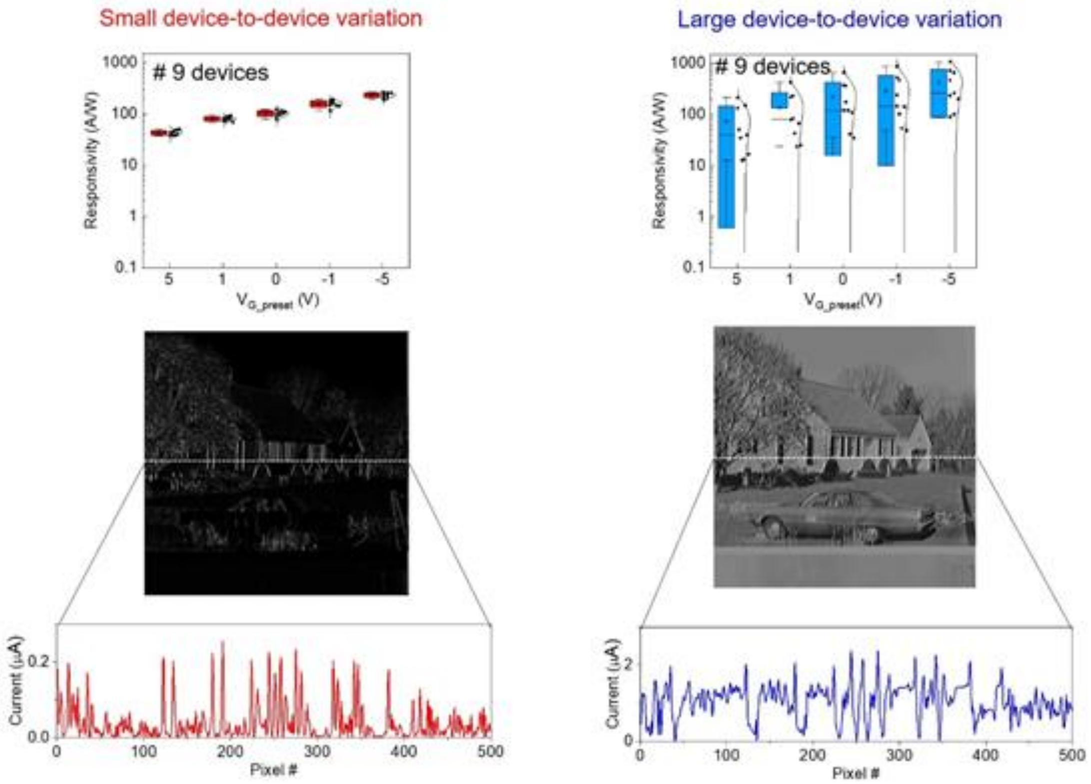

Figure S13. Comparison of the edge enhancements' effectiveness between two different arrays exhibiting small and large device-to-device variation.

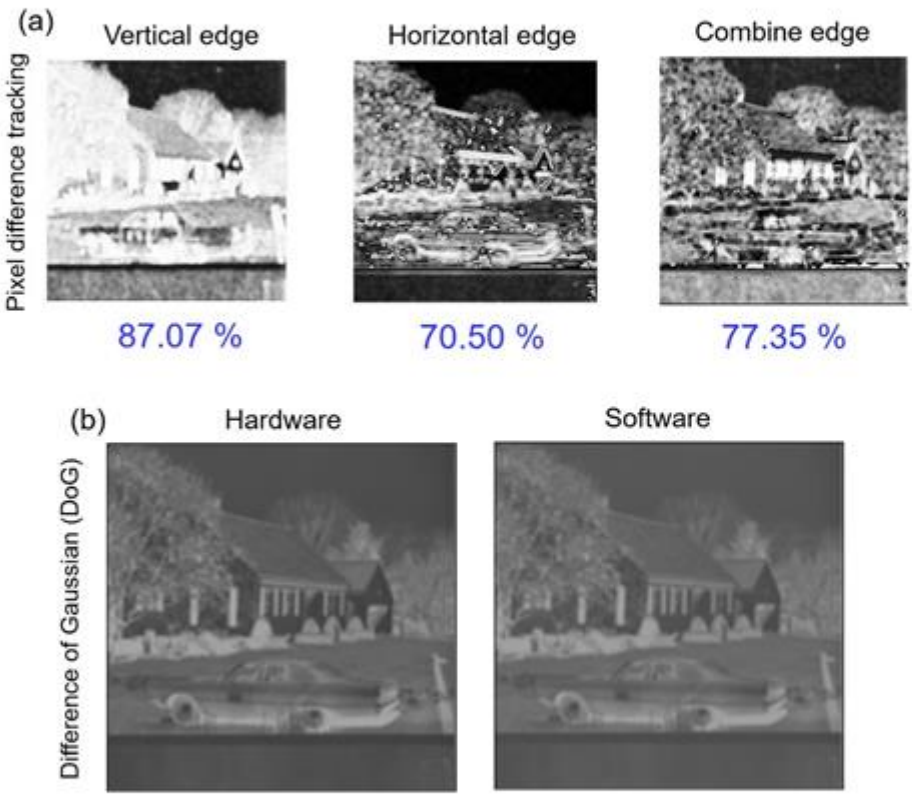

Figure S14. (a) Pixel-to-pixel difference tracking results in the structure similarity score, which evaluates the accuracy of in-sensor computed and software-simulated images. (b) Feature enhancement via hardware and software-based convolution by DoG kernel.

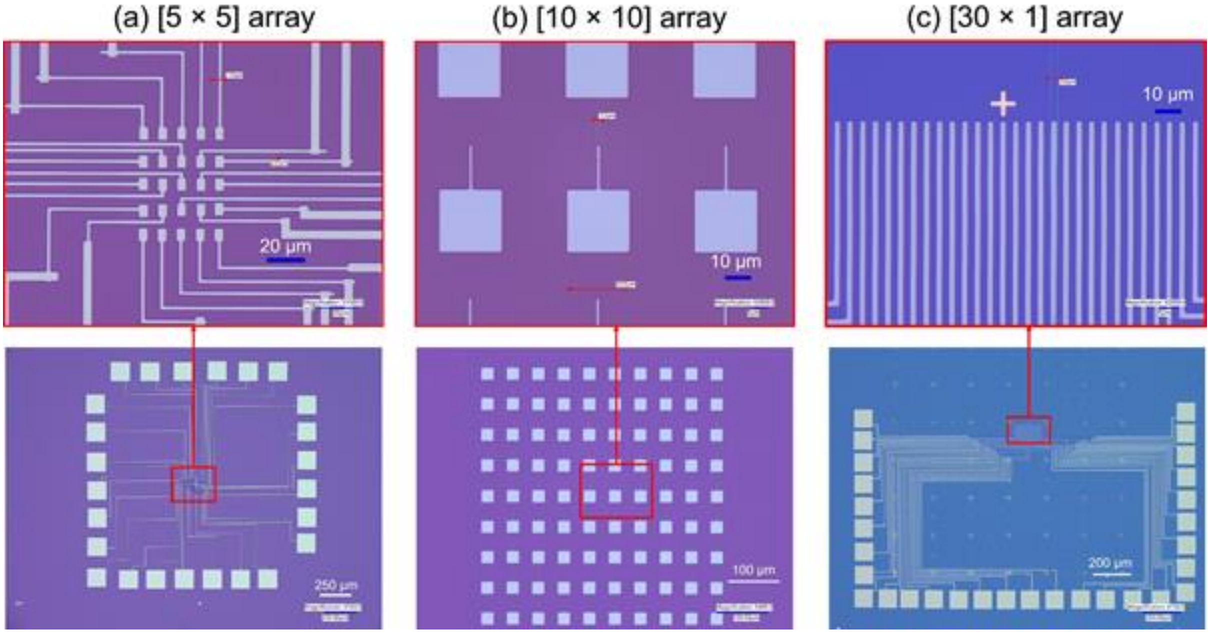

Figure S15. Different array configurations are approached by the metal-etching method.

References

[1] C. Choi, J. Leem, M. Kim, A. Taqieddin, C. Cho, K. W. Cho, G. J. Lee, H. Seung, H. J. Bae, Y. M. Song, *Nature communications* **2020**, 11, 5934.

[2] L. Mennel, J. Symonowicz, S. Wachter, D. K. Polyushkin, A. J. Molina-Mendoza, T. Mueller, *Nature* **2020**, 579, 62.
